# Differential microstructural alterations in rat cerebral cortex in a model of chronic mild stress depression

**DOI:** 10.1371/journal.pone.0192329

**Published:** 2018-02-12

**Authors:** Ahmad Raza Khan, Christopher D. Kroenke, Ove Wiborg, Andrey Chuhutin, Jens R. Nyengaard, Brian Hansen, Sune Nørhøj Jespersen

**Affiliations:** 1 Center of Functionally Integrative Neuroscience, Aarhus University Hospital, Aarhus, Denmark; 2 Advanced Imaging Research Center, Oregon Health & Science University, Portland, Oregon, United States of America; 3 Core Center for Molecular Morphology, Section for Stereology and Microscopy, Centre for Stochastic Geometry and Advanced Bioimaging, Aarhus University, Aarhus, Denmark; 4 Department of Physics and Astronomy, Aarhus University, Aarhus, Denmark; University of Pennsylvania, UNITED STATES

## Abstract

Chronic mild stress leads to depression in many cases and is linked to several debilitating diseases including mental disorders. Recently, neuronal tracing techniques, stereology, and immunohistochemistry have revealed persistent and significant microstructural alterations in the hippocampus, hypothalamus, prefrontal cortex, and amygdala, which form an interconnected system known as the stress circuit. Most studies have focused only on this circuit, however, some studies indicate that manipulation of sensory and motor systems may impact genesis and therapy of mood disorders and therefore these areas should not be neglected in the study of brain microstructure alterations in response to stress and depression. For this reason, we explore the microstructural alterations in different cortical regions in a chronic mild stress model of depression. The study employs ex-vivo diffusion MRI (d-MRI) to assess cortical microstructure in stressed (anhedonic and resilient) and control animals. MRI is followed by immunohistochemistry to substantiate the d-MRI findings. We find significantly lower extracellular diffusivity in auditory cortex (AC) of stress groups and a significantly higher fractional anisotropy in the resilient group. Neurite density was not found to be significantly higher in any cortical ROIs in the stress group compared to control, although axonal density is higher in the stress groups. We also report significant thinning of motor cortex (MC) in both stress groups. This is in agreement with recent clinical and preclinical studies on depression and similar disorders where significant microstructural and metabolic alterations were found in AC and MC. Our findings provide further evidence that the AC and MC are sensitive towards stress exposure and may extend our understanding of the microstructural effects of stress beyond the stress circuit of the brain. Progress in this field may provide new avenues of research to help in diagnosis and treatment intervention for depression and related disorders.

## Introduction

Chronic mild stress (CMS) is a major cause of illness, in many cases leading to depression with destructive effects on the life of individuals of all ages [1, 2]. Diagnosis of depression relies exclusively on behavioral symptoms, such as anxiety, excessive feelings of guilt, lethargy, anhedonia, insomnia, to name a few. The persistence and intensity of these symptoms are used to determine depression severity following the Diagnostic and Statistical Manual of Mental Disorders (DSM) V guidelines[3]. Despite decades of intense preclinical and clinical investigation, the neurobiological basis of depression remains unclear [4, 5]. Clinical and preclinical studies have established that depression likely arises from dysfunction in cortical and subcortical regions, mainly prefrontal cortex, hippocampus, hypothalamus, amygdala and caudate putamen, also known as the stress circuit of the brain [6–11].

Most studies have focused on this stress circuitry, although some studies indicate that manipulation of sensory and motor systems may impact genesis and therapy of mood disorders and therefore should not be neglected [12–14]. Significant microstructural changes in cerebral cortical regions relevant to mood disorders have been found in animals subjected to visual and auditory fear conditioning, as well as in animal models of emotional stress [12, 15–18]. The underlying microstructural alterations may be related to the formation and retraction of dendrites and synaptic structures which can happen rapidly in sub-populations of cortical neurons during various sensorimotor learning experiences [19]. Such information extends our understanding of depression beyond the stress circuitry of the brain by including cortical regions highly connected to the typically studied limbic regions [20]. Postmortem studies of major depressive disorder using stereology and neuronal tracing techniques have demonstrated lower density of glial cells and reduction in neuronal cell size in stress sensitive subcortical and cortical regions [21–23] and similar findings have been reported in animal models of depression [24, 25]. Such microstructural alterations may be associated with some of the characteristics of depression such as attention deficit, anxiety, cognitive impairment and/or memory loss. Clearly, stereology and neuronal tracing techniques contribute significantly to our understanding of depression and similar disorders at the microstructural level, but these techniques are not suitable for clinical use due to their invasive nature.

Unfortunately, traditional diffusion MRI (d-MRI) parameters are non-specific (e.g. diffusion tensor parameters) and often difficult to interpret. More recent strategies combining d-MRI with biophysical modeling improve specificity by providing microstructural parameters. Examples of these are neurite density, longitudinal intra-neurite diffusivity, axonal water fraction, and intra- and extra-axonal diffusivity [26–30]. While validation of compartmental diffusivities remains challenging, other model parameters such as the neurite density lend themselves to validation against histology [27, 31, 32]. Recent work has characterized multiple aspects of gray matter cellular-level changes with d-MRI, such as significantly higher neurite density in amygdala using biophysical modeling of d-MRI data and histology in a CMS exposed rat model of depression [25]. However, for precise computation of these model parameters, large datasets are required, which mostly limits the application of model parameters to fixed tissue preparations. A less specific, but clinically feasible, d-MRI technique is diffusion kurtosis imaging (DKI) [33], which has been demonstrated to be sensitive to subtle microstructural alterations in both clinical [34–36] and preclinical disease models [11, 25, 37, 38]. However, DKI parameters are statistical characteristics of the d-MRI signal and are not defined in terms of tissue microstructure, which prevents direct histological validation of DKI parameters. This limitation may be circumvented by exploring DKI and biophysical modeling together supplemented with quantitative histology. This comparative approach holds the potential to provide a clearer interpretation of the clinically feasible DKI methods in terms of tissue specific parameters obtained from the biophysical model and histology. In this manner, d-MRI methods provide a strong basis not only for improving our understanding of the basic neurobiology of stress and depression but also for clinical studies of these conditions.

The present study used d-MRI data obtained previously [25] to address the specific role of microstructural alterations in sensory and motor cortical regions of a CMS rat model of depression [39, 40]. Specifically, we target the motor cortex (MC), somatosensory cortex (SC), auditory cortex (AC) and visual cortex (VC). We investigate these regions in anhedonic, resilient and control animals (see Methods for details on these groups). To our knowledge, in spite of the suggested role of the cortex in depression [17, 41, 42] no study has investigated microstructural alterations in these cortical regions of an unpredictable CMS model of depression. Our study explores microstructural alterations using d-MRI analyzed with both biophysical modeling and DKI. To corroborate the d-MRI findings, immunohistochemistry was performed on a previously fixed tissue sections to perform quantitative histology. Immunohistochemistry was performed using MAP2 (a dendritic marker) and NF-H (an antibody for mature axons) to expose changes in dendrites and axons in the targeted ROIs. Cortical thickness was also measured in these ROIs using histological montages from all three groups.

The study reveals a significant reduction in extracellular diffusivity (*D*_eff_) in AC of both stress groups (anhedonic and resilient), while the diffusion tensor parameter, FA showed significantly higher in the MC of the resilient group. Histological analysis of axonal density and cortical thickness analysis corroborated the d-MRI findings. Demonstration of such microstructural alterations may be useful for interpretation of behavioral changes associated with auditory and visual fear conditioning paradigms. Furthermore, the findings may aid in generating new hypotheses about CMS and depression, and provide new target regions for clinical monitoring of disease.

## Materials and methods

Samples and MRI data employed in this study were reported in [25, 43]. Additional method details and raw MRI and histological data are available online [43] and also described briefly here in the following sections.

### Animals

Adult male Wistar rats (Taconic, Denmark) were randomly exposed to an array of unpredictable mild stressors for 8 weeks to drive the animals into depression [39]. Following the unpredictable CMS paradigm, rats were segregated into anhedonic (N = 8) or resilient (N = 8) groups based on individual sucrose consumption. An age-matched group of animals, unexposed to stressors, served as control (N = 8). After the stress paradigm, all animals were euthanized by exsanguination using isotonic saline containing heparin (10 IU/mL) followed by transcardial perfusion fixation using 4% buffered paraformaldehyde (pH 7. 4). The isolated brains were then immersion fixed in fresh paraformaldehyde solution for weeks prior to the MRI experiments. Animal handling and all experimental procedures were performed in accordance with the national guidelines for animal research and with permission from the Animal Experiments Inspectorate of the Danish ministry of Food, Agriculture and Fisheries, Denmark (2013-15-2934-00814).

### Imaging protocol

For each brain, the left hemisphere was isolated and scanned. Prior to MRI, each sample was washed with phosphate buffered saline (PBS) for 48 hours to remove paraformaldehyde and to minimize associated T2-related signal attenuation. Samples, where perfusion had failed or where the sample was physically damaged are excluded from the data analysis. Subsequently, sample was placed in an MRI compatible tube filled with a magnetic susceptibility matched fluid (Fluorinert FC-40,3M Zwijndrecht, Belgium). MRI data were acquired on 9.4T Bruker Biospec preclinical MRI system (Bruker Biospin, Germany) with a 15 mm bore mounted quadrature volume coil at room temperature (21°C). Sample temperature was not monitored independently during the scans, although a consistent ADC value of lateral ventricle regions indicates stable sample temperature during the long ex-vivo d-MRI scans. Diffusion data acquisition was performed with standard diffusion spin echo preparation at 250 μm isotropic resolution with 12 fixed directions and 14 b-values (b = 0, 0.5, 1.0, 1.5, 2.0, 2.5, 3.0, 3.5, 4.0, 4.5, 5.0, 6.0, 7.0, 8.0 ms/μm^2^) with the following parameters: TR/TE = 6500 ms/26 ms, Δ/δ = 15/5 ms, field of view (FOV) 25. 5 × 12. 5 mm and matrix size 102 × 50. Corresponding anatomical images were also acquired using a rapid acquisition with relaxation enhancement (RARE) pulse sequence with the following parameters: TR/TE = 3500/11 ms, averages = 16, rare factor = 8, BW = 46.9, in-plane resolution = 62. 5μm and slice thickness = 250 μm with no slice gap. The scan time of a sample for the anatomical images was approximately 29 minutes.

### Parameter estimation

All d-MRI data sets were analyzed in a voxel-wise manner using the nonlinear least squares Levenberg-Marquardt algorithm as implemented in Matlab (The Mathworks Inc., Natick, MA). The neurite density model assumes that the d-MRI signal has contributions from two non-exchanging tissue compartments: 1) Sc, describing diffusion in axons and dendrites (collectively termed neurites) and 2) Si, extra neurite diffusion signal. It is described in greater detail elsewhere. (Jespersen et al., 2010; Jespersen et al., 2007). From the neurite density model, three parameters were considered in this study: neurite density, longitudinal intra-neurite diffusivity (*D*_L_) and extracellular diffusivity (*D*_eff_). DKI estimation was performed using a conventional non-linear least squares fitting procedure on a subset of the diffusion data consisting of all shells in the b-value range 0–4.5ms/μm^2^. Kurtosis parameters considered were: mean kurtosis (MK) [33], axial kurtosis (AK) [44], radial kurtosis (RK), mean of kurtosis tensor (MKT) [45, 46], axial tensor kurtosis (*W*_L_) and radial tensor kurtosis (*W*_T_) following the definitions in [47]. The diffusion kurtosis tensor captures the leading deviations from Gaussian diffusion. We recently proposed a fast kurtosis method [45, 47] enabling accurate estimation of mean, radial, and axial kurtosis on the basis of reduced data (fewer directions) compared to conventional diffusion kurtosis imaging [33]. A recent review paper provides an overview of the fast kurtosis methods ([48]. The kurtosis tensor method requires just two minutes of scan time [47] and the Matlab code to extract the kurtosis metrics is available at https://github.com/sunenj/Fast-diffusion-kurtosis-imaging-DKI. Two important metrics derived from the diffusion tensor were also included, namely mean diffusivity (MD) and fractional anisotropy (FA) [49, 50]. Procedures for neurite density model fitting and DKI parameter estimation are detailed in [25].

**Fig 1:**
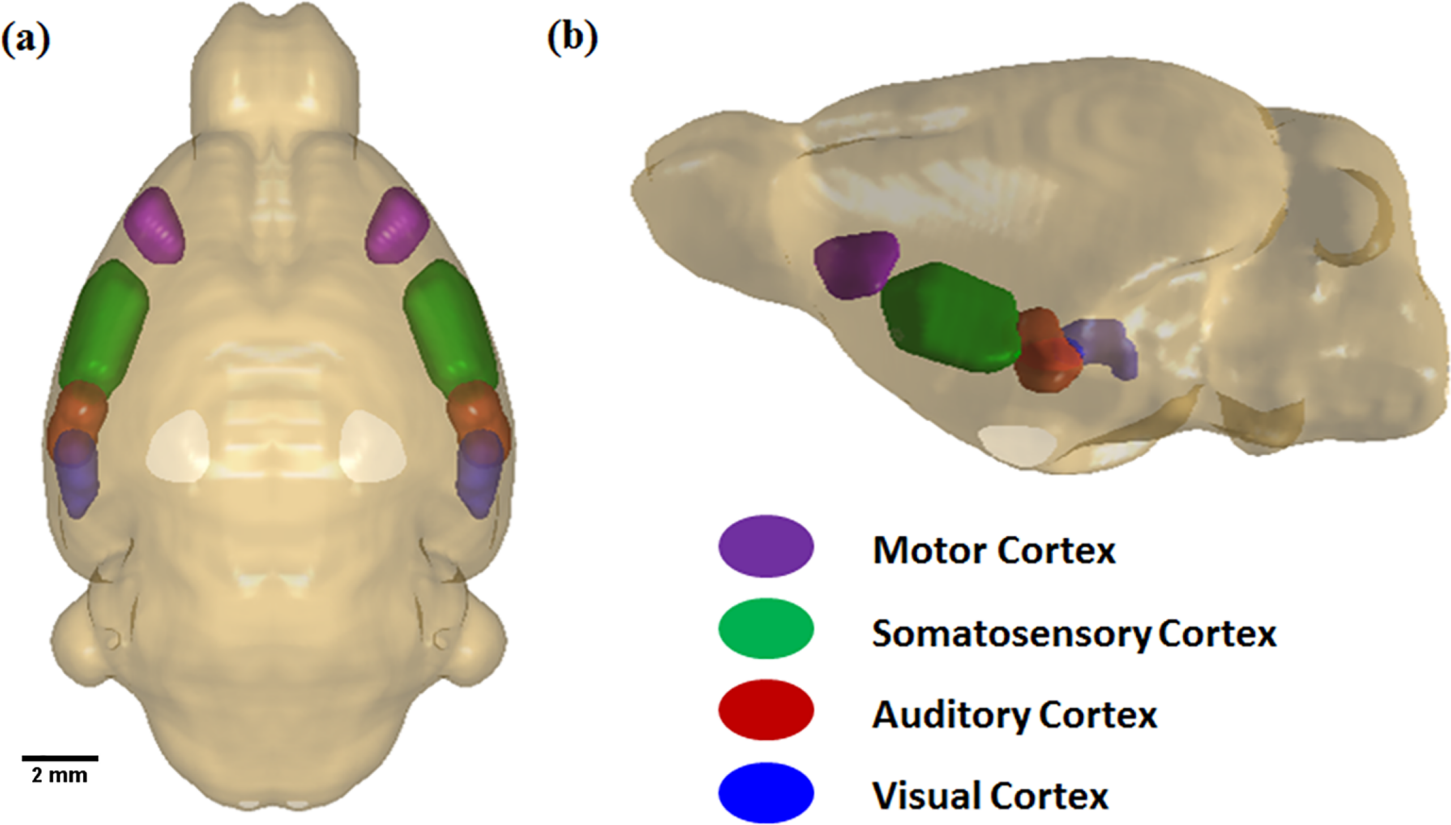
A representative 3D dorsal view of the brain. Region of interest (ROIs) intersect sub-regions of motor cortex: violet, Somatosensory cortex: green, Auditory cortex: red and Visual cortex: blue.

**Fig 2:**
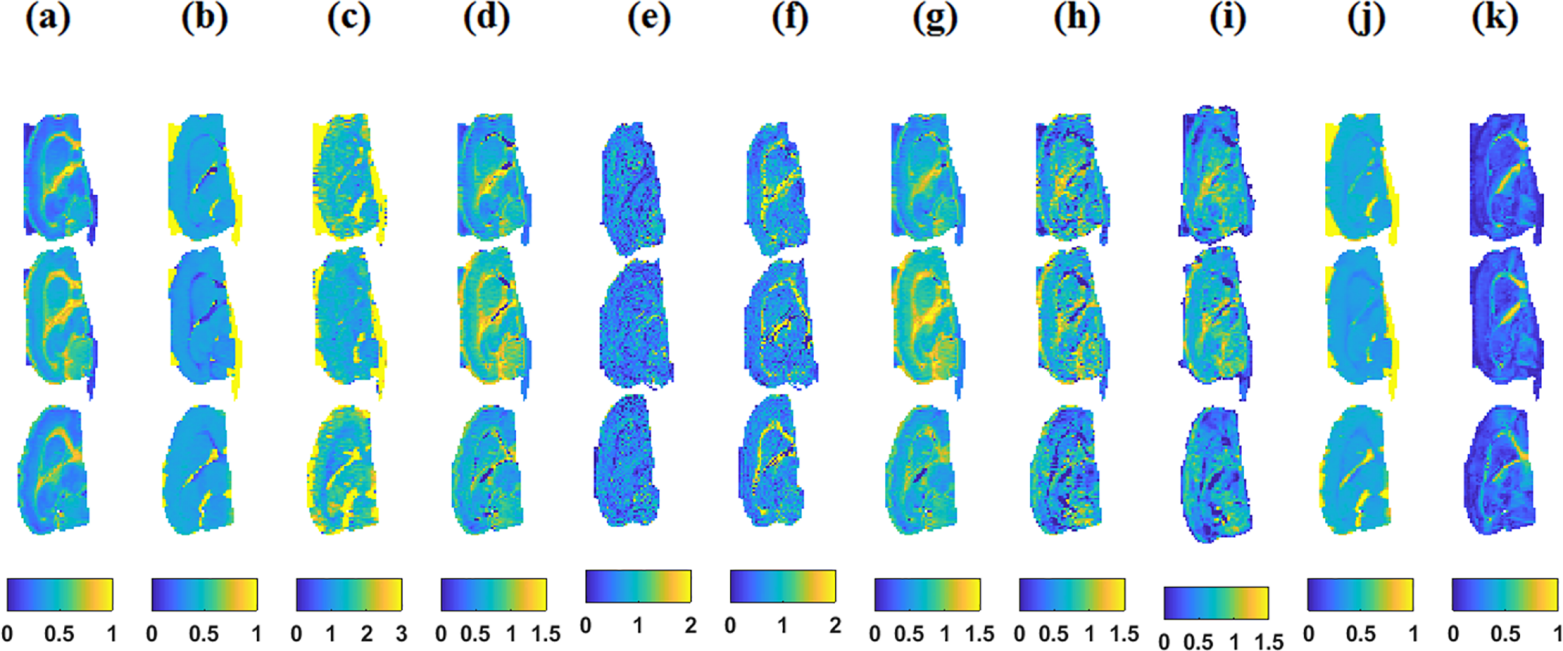
Top row: Control; middle: Anhedonic; lower: Resilient. (a)-(c) shows neurite density model parameters: neurite density (Neu), extracellular diffusivity (*D*_eff_) and longitudinal intra neurite diffusivity (*D*_L_), (d)-(f) shows kurtosis parameter: mean kurtosis (MK), axial kurtosis (AK), and radial kurtosis (RK), (g)-(i) mean of kurtosis tensor (MKT), axial kurtosis tensor (*W*_L_) and radial kurtosis tensor (*W*_t_), (j)—(k) shows mean diffusivity (MD) and fractional anisotropy (FA) maps.

Prior to analysis, the MRI data were inspected visually for quality (artefacts, sample damage etc). During this inspection, the *T*_2_ weighted anatomical images showed that two brains from the anhedonic group had been damaged during preparation. Similarly, it was found that perfusion fixation had failed in one of the control group animals. These samples were excluded from the study. Brain sectioning was performed in the horizontal plane as described previously [25]. Sections between -3.10 and -4.10 mm ventral to the Bregma intersect cortical areas that serve motor, somatosensory, auditory, and visual functions. The specific cortical areas defined in the Paxinos and Watson atlas that were included in the regions of interest (ROIs) used in this study are MC:(Primary (M1) and secondary motor cortex (M2)), SC: (Primary somatosensory cortex barrel field), AC: (Primary auditory cortex (Au1) and Dorsal auditory cortex (AuD)), and VC: (Visual cortex (V2L) and temporal association cortex). Four corresponding ROIs were also manually delineated (MC, SC, AC, and VC) (Fig 1) on the d-MRI images in a blinded way with reference to a rat brain atlas [51]. As diffusion data was acquired in the coronal plane and the reference coordinates for the cortical region in the horizontal plane of the brain atlas started from (bregma—3.10 mm, and interaural 6.90 mm), the parametric maps of d-MRI were extracted from the ROIs in between coordinates (Bregma -3.10 to Bregma -4.10) in the horizontal sections to match the histological data (Fig 2).

### Histology

Following MRI, the sample was rinsed with PBS and stored in paraformaldehyde solution until sectioning. Before tissue sectioning (Vibratome 3000, Vibratome Co., St. Louis, MO) the hemisphere was again rinsed with PBS and embedded in 5% agar solution prepared in PBS. All brains were cut horizontally into 60 μm thick sections, immersed in fixative and placed at -20°C prior to staining. Brain tissue sections within the co-ordinates (Interaural 5. 9–6. 9 and Bregma -3. 10 to -4. 10) were selected for tissue staining, as they contain all the targeted ROIs.

#### Axonal (neuro-filament) and dendritic (MAP2) immunohistochemistry

One set of tissue sections underwent immunostaining with the anti-microtubule associated protein 2 (*MAP2*) (AbCam, Cambridge, UK) a neuronal cell body and dendritic marker, and another set of the tissue sections with anti-neuro-filament 200 antibody (NF-H) (AbCam, Cambridge, UK), an axonal neurofilament marker for mature neurons. Prior to staining, sections were rinsed with 1X tris-buffered saline (TBS). Endogenous peroxidase activity was quenched with a cocktail of TBS, methanol, and H_2_O_2_ (3%) for 15 minutes. Tissue sections were subjected to heat mediated antigen retrieval with target retrieval solution buffer (Dako, Denmark, S1699) at 80°C for 30 minutes. Thereafter, each section was rinsed three times with TBS (pH 7. 4) and incubated with the blocking buffer for 30 minutes before applying the primary antibody. The tissue sections were stained with *MAP2* (antibody, 1:500) and NF-H (1:500) overnight at 4°C and subsequently diluted with horseradish-peroxidase (HRP) coated secondary antibody (1:200) (Dako, Glostrup, Denmark) in a TB buffer containing 1% bovine serum albumin (BSA). Antibodies were detected using the HRP complex, and labeling was revealed after incubating the sections in 3,3′-diaminobenzidine (DAB) peroxidase solution (31. 5μl DAB, 1 μl H_2_O_2_ in 1. 6 ml 0. 01 M TBS, pH 7. 2) for 5 min followed by washing and counterstaining with a nuclear stain. Finally, tissue sections were dehydrated in a series of ethanol concentration and subsequently treated with xylene before mounting with a permanent mounting medium on superfrost+ glass slides (Fisher Scientific, Denmark).

### Light microscopy

**Fig 3:**
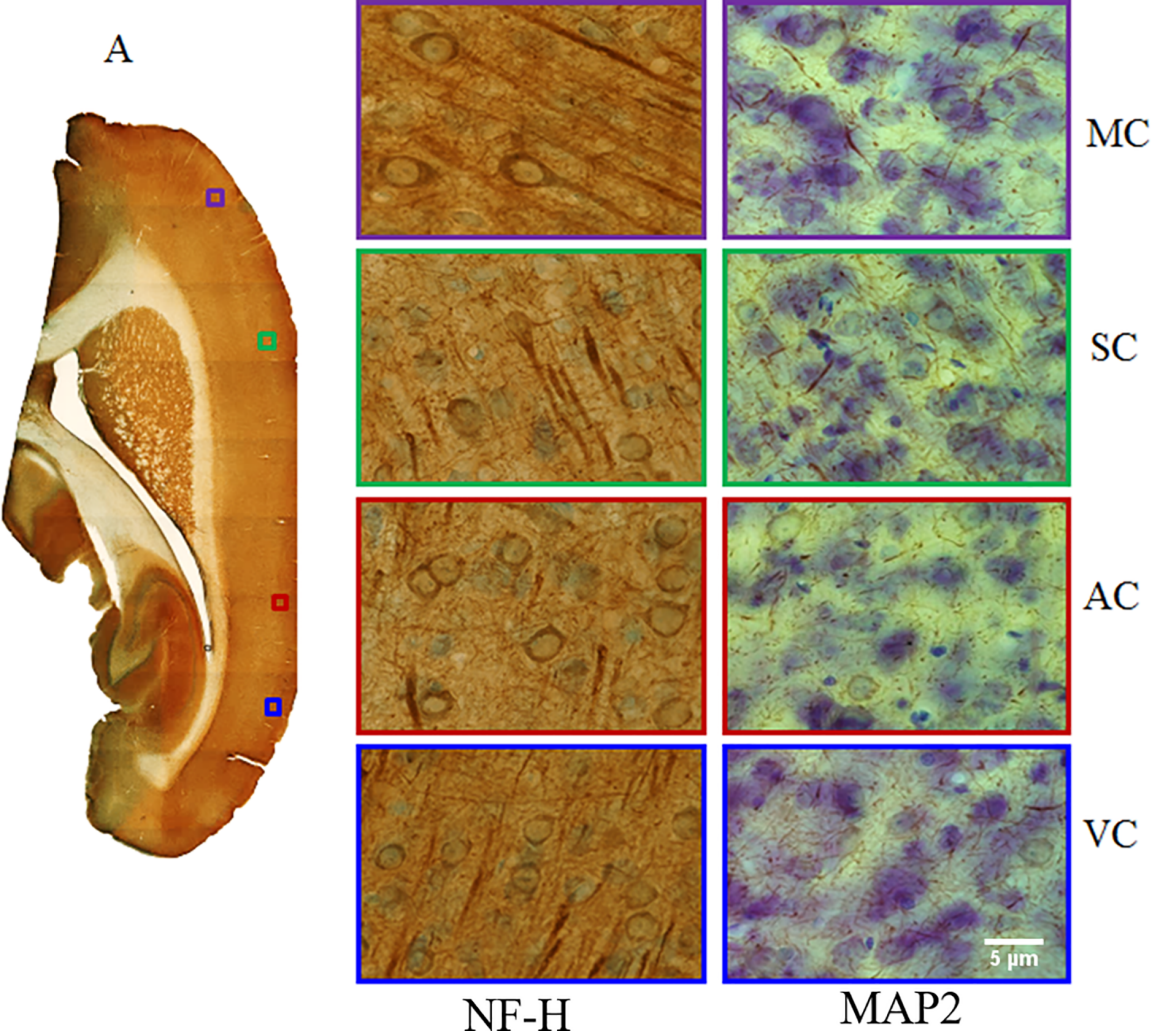
(A) Montage of the whole tissue section acquired with 4x objective lens and ROIs (Violet: motor cortex (MC), green: somatosensory cortex (SC), red: auditory cortex (AC), and blue: visual cortex (MC). (B) Immunohistological image of neuronal filament using axonal antibody (NF-H) and (C) immunohistological image of neuronal dendrites using antibody (MAP2) acquired using a light microscope with a 63x objective lens.

Immunohistological sections were imaged with an Olympus BX51 microscope (Olympus Inc., Tokyo, Japan). Whole tissue section montages were acquired with a 4x objective lens, and high-resolution images were acquired with a 63x oil objective lens (Fig 3). Systematically sampled fields of views (FOVs) of each section were taken within the MC, SC, AC and VC regions of the brain. Images were imported in ‘tif’ format for further quantitative analysis in Matlab (The Mathworks, Natick, MA).

### Image processing and analysis

The microscopic image analysis was performed automatically using identical contrast enhancement and image operation settings in Matlab for all images. Scaling for luminosity was performed as described in recent publications [25, 38] after which contrast enhancement for tissue microstructure was performed allowing quantification of the axonal and dendritic microstructure using Matlab's image processing toolbox.

#### Axonal density [%]

**Fig 4:**
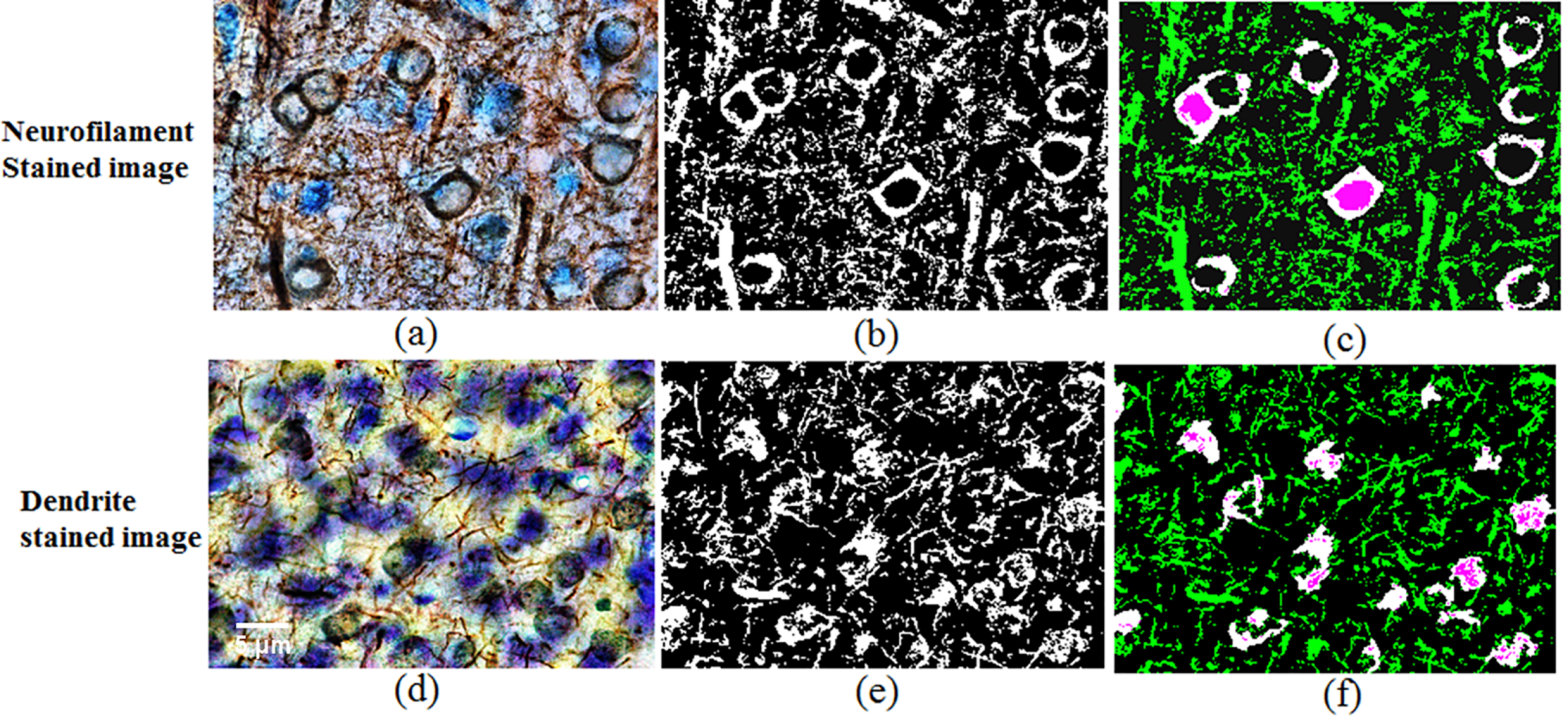
(a, d) Contrast enhanced image, (b, e) binary image of contrast enhanced image and (c, f) binary image of contrast enhanced image (green) with cell body mask (white and pink) using image processing tool in Matlab. The Cell body mask was subtracted from the contrast enhanced a binary image to calculate the axonal and dendritic fraction of the image.

To estimate the axonal density from the immunostained images (NF-H), a previously [38] described Matlab based quantitative histological approach was applied. The images were imported and contrast enhanced for optimal neurofilament detection (Fig 4A). Images were then thresholded to 30% relative to the signal level and converted into a binary image (Fig 4B). The NF-200 antibody binds to a high molecular weight neurofilament subunit present in axons and in neuronal cell bodies. To estimate the axonal density precisely, the cell bodies must, therefore, be removed from the binary map. This was achieved by extracting immunostained neuronal cell bodies in a separate binary image produced using the function ‘bwpropfilt’ (part of Matlab's image processing toolbox). This function extracts objects from a binary image using image properties such as ‘eccentricity’ and ‘equal diameter’. The cell bodies identified in this manner were then subtracted from the contrast enhanced a binary image of NF-H binding sites (containing both axons and cell bodies) (Fig 4C). All remaining pixels were considered axonal components and the axonal density [%] was then obtained by dividing by the total number of image pixels.

#### Dendritic density [%]

The dendritic density was estimated from immunostained (MAP2) images, in a workflow similar to the one described for axonal density estimation. Here too, an image contrast enhancement protocol was applied, this time fine-tuned for dendrites (Fig 4D). The contrast enhanced images were converted into binary images (Fig 4E) from which contributions of cell bodies and/or nuclei (estimated as above) were subtracted (Fig 4F). Remaining pixels were identified as dendritic components from which an estimate of the dendritic density [%] was calculated in the same way as the axonal density.

### Cortical morphometry

For cortical thickness measurement, three or more montages were selected between the coordinates (Bergama -3.10 to -4.10) from each animal and 5 lines were drawn on each ROI of a tissue montage Histological montages were imported in ImageJ [52]. Lines were drawn with freehand line tool of Image J on the cortical surface as perpendicular as possible to the pial surface and systematically on each ROI [53]. Five straight line measurements were obtained from each ROI, using the 'analyze' function of Image J. Thickness measurements were then exported for further statistical analysis.

Numerical value of all the histological data is also presented in S1 File (S1_File.xlsx) on page 13.

### Statistical analyses

Diffusion MRI and histological data were separately fit in Matlab to a linear mixed effect model with animals as random effects and group as a fixed effect, as in [25]. Significant differences between groups were identified using an F-test with a 5% level of significance. Degrees of freedom were computed using the Satterthwaite approximation (Satterthwaite, 1946). If significant, subsequent (post-hoc) pairwise tests were performed and FDR correction [54] was applied for multiple testing comparisons. Confidence intervals (CI) (95%) were also generated as output, to provide an estimate of fixed effect size and variability [55]. When applicable, graphs report CI and estimated means.

## Results

**Fig 5:**
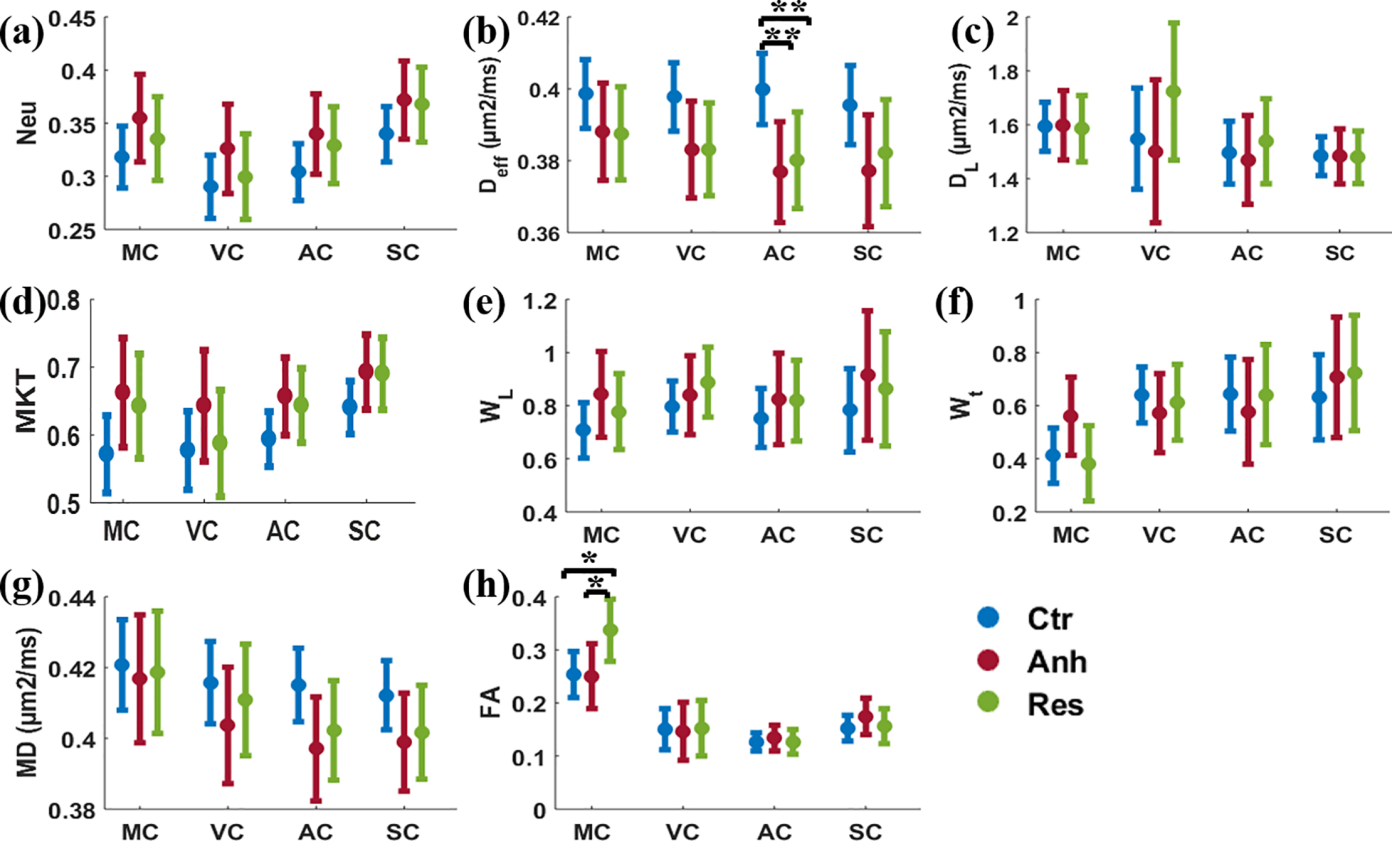
(a) Neurite density, (b) extracellular diffusivity (*D*_eff)_ (μm^2^/ms), (c) intra neurite diffusivity (*D*_L_) (μm^2^/ms), (d) mean of kurtosis tensor (MKT), (e) axial kurtosis tensor (W_L_), (f) radial kurtosis tesnsor (W_t_), (g) mean diffusivity (MD) and (h) fractional anisotropy (FA) data as mean ± confidence interval (CI) from MC, VC, AC and SC regions of the brain from control, anhedonic and resilient group. Linear mixed model regression analysis was performed in Matlab. Significantly lower *D*_eff_ was observed in AC of the anhedonic and the resilient group (** p<0.01) in comparison to control while FA, shows significantly higher (* p<0.05) in MC of resilient group in comparison to control.

All d-MRI based parameters are represented in Fig 5A–5H for all ROIs analyzed in this study. Diffusion parameters are reported separately as neurite density model parameters, traditional kurtosis parameters (S1 Fig), tensor based kurtosis parameters and diffusion tensor parameters.

### Neurite density model parameters (Neu, *D*_eff,_ and *D*_L_)

Significant reduction in the neurite density model parameter, *D*_eff_ was found in AC in both the stress groups in comparison to control (p = 0. 009) (Fig 5B). Reduction in *D*_eff_ in other ROIs was also observed, although not significant. The remaining biophysical model parameters considered here (Neu and *D*_L_) did not reveal significant alterations in any cortical region under investigation (Fig 5A and 5C).

### Traditional kurtosis parameters (MK, AK, and RK)

There was no significant alteration in any kurtosis parameter in any ROIs analyzed. However, MK showed a similar pattern of as neurite density in all ROIs. Closer scrutiny shows a consistent pattern of higher MK, in both the stress groups in all four ROIs, similar to the variation observed for neurite density (S1A–S1C Fig).

### Tensor based kurtosis parameters (MKT, *W*_L,_ and *W*_T_)

Tensor based kurtosis parameters have not shown any significant alteration in any ROIs (Fig 5D–5F). Only *W*_T_ showed marked increase in the MC, however, could not survive multiple comparison tests (p1 = 0.072, between control and anhedonic and p3 = 0.056 between anhedonic and resilient group) (Fig 5F). MKT showed a higher value in the MC (p = 0. 07) and other ROIs as well, those reached only trend-level statistical significance (Fig 5D)).

### Diffusion tensor parameters (FA and MD)

Similar to the *D*_eff_ changes in the neurite density model parameter, AC also showed significantly lower MD in the anhedonic group, however, could not survive multiple testing corrections (p = 0. 056) in comparison to control (Fig 5G). Lower MD was also observed in VC and SC although not significant in comparison to control. MC showed significantly higher FA in the resilient group only in comparison to control (p = 0. 012), while other ROIs showed no significant alteration in FA in comparison to control (Fig 5H). There were no significant alterations in any ROIs using axial and radial diffusivity parameters and are not included in the study.

Similar to the neurite density model parameters and kurtosis parameters, FA and MD have broader CI in all the ROIs of both the stressed groups.

### Histological axonal and dendritic density [%]

**Fig 6:**
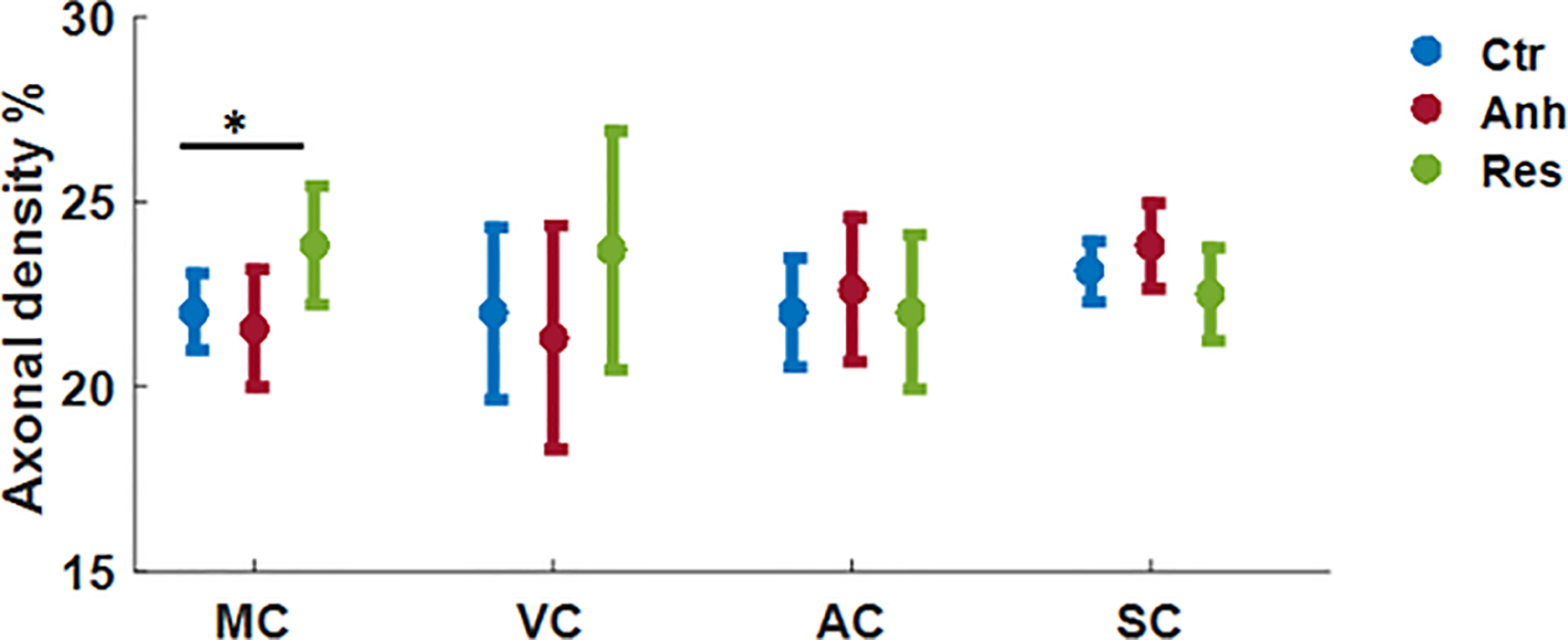
Axonal density [%], data as mean ± confidence interval (CI) from MC, VC, AC and SC regions of the brain from control, anhedonic and resilient group. Linear mixed model regression analysis was performed in Matlab. Significantly higher axonal density [%] was observed in MC of resilient group (* p<0.05) in comparison to the anhedonic group.

In the histological analysis, only MC showed significantly higher axonal density (p = 0.04) and only in the resilient group in comparison to the control (Fig 6). There were no other significant alterations in axonal density in any ROIs investigated. Dendritic density did not show any significant alterations in any of the four ROIs investigated (S2 Fig).

### Cortical morphometry

**Fig 7:**
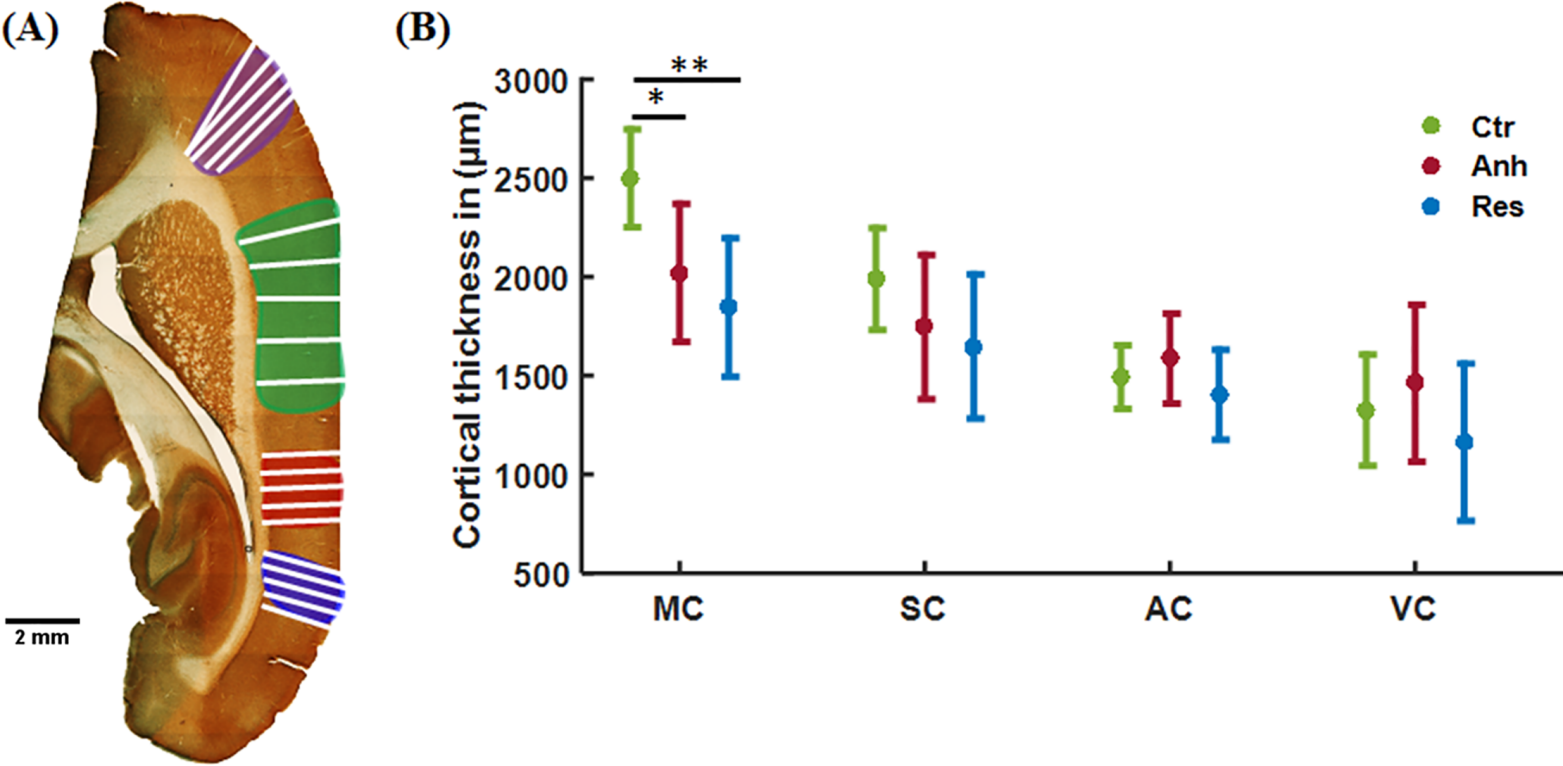
Cortical thickness measurement using straight line tool on image J. An equal number of lines were manually drawn on each ROIs on a histological montage (a). CI (95%) and mean of the cortical thickness of all the ROIs from the control, anhedonic and resilient group (b). Only MC showed significantly lower thickness in both the stress groups (*p<0. 05, **p<0. 01).

Cortical thickness measurement as shown in Fig 7A demonstrated significantly lower thickness in the MC of the anhedonic (*p = 0.012) and resilient group (** p = 0.006) in comparison to control (Fig 7B). SC showed markedly low thickness, similar to MC in the stressed group, but not significantly different from controls. However, the CI of the thickness in the stressed groups is broader than in controls, a similar trend as with MRI and immunohistological data.

Although statistically significant differences were observed between animal groups in cortical morphometry and axonal fraction in the MC, and this supports the findings of differences in d-MRI based results, statistically significant correlations were not found between any of the d-MRI parameters and histological parameters computed in this study.

## Discussion

The unpredictable CMS animal model of depression investigated here is considered one of the most realistic models of depression with the demonstrated face, predictive, etiological and construct validity [39, 56, 57]. This model has been investigated using neuronal tracing techniques, stereology and immunohistochemistry, and has demonstrated significant microstructural alteration in brain regions, such as hippocampus [25, 58–60], prefrontal cortex [61, 62], amygdala [25, 63, 64] and caudate putamen [63]. However, microstructural alterations in cortical regions except for prefrontal cortex have not previously been subjected to investigation. Evidence of significant microstructural remodeling was found here as reflected in the diffusivity parameters in AC where *D*_eff_ was found to decrease significantly in both stress groups. These findings are in agreement with other studies where evidence of stress induced remodeling in AC has also been found albeit using other methods. A recent preclinical MRI study using voxel based morphometry (VBM) reported an increased volume of AC in animals exposed to an auditory fear conditioning paradigm. Moreover, histology in that study showed higher neurite and spine density in AC with a significant correlation to VBM from AC [65].However, in the present study there were no significant alterations in neurite density or any of the fast kurtosis parameters in all targeted ROIs in the stressed brain in comparison to control, and no significant correlation of d-MRI parameters and histology. Nonetheless, histological data support the significant increase in FA in the MC of the resilient group as significant increase in axonal fraction may have contributed to the higher anisotropy [66, 67]. The significant finding of lower *D*_eff_ in the stress group, however, might be due to alterations in synaptic remodeling [68], axonal sprouting, and synaptogenesis [69, 70]. A similar effect was found in our recent study [25] where significant reduction in *D*_eff_ were also found in the amygdala, hippocampus and caudate putamen of the stressed rats in comparison to control. This is an important finding because it contradicts the popular assumption of constant compartmental diffusivities in most NODDI implementations [29].

Besides microstructural alterations, a micro-PET based study [71] reported hyperactive glucose metabolism in AC of the rat brain exposed to the unpredictable CMS paradigm. The hyperactive AC after unpredictive CMS exposure also supports the present findings indirectly, as the tendency for elevated neurite density seen here might be an indication of activated AC in both stress groups. Another preclinical study using a different stress model has revealed significantly higher c-fos expression (an indirect marker of neuronal activity) in primary AC and secondary SC in a physical stress model (foot shock stress), and in the temporal association cortex in a psychological stress model [18]. Although the present study has not shown any significant alteration in SC, lower *D*_eff_ and MD are apparent in SC of stress groups and the higher (non-significant) neurite density in SC of both the stress groups is consistent with lower diffusivity.

Apart from preclinical studies, several clinical studies have reported reduced gray matter volume in depression and similar disorders [72–75]. Postmortem studies using stereology and immunohistochemistry have also reported reduced neuronal cell size and glial cell numbers in major depressive disorder [21–23] in agreement with the results from preclinical studies showing substantial glial atrophy in depression and similar disorders [20, 24]. Such reduction in gray matter volume may partly explain the higher MR based neurite density observed in all the ROIs of the present study. The estimation of astrocyte density could support such speculation, although the absence of astrocyte immunohistochemistry is a limitation of the present study.

Different microstructural responses have been reported for different stress paradigms applied. For instance, dendritic atrophy in AC was reported after different chronic stress exposure paradigm [13, 18, 76]. Moreover, Bose et al. (2010) and Yu et al. (2015) did not find any significant microstructural alterations in VC following chronic restraint stress and a physical and psychological stress paradigm. In contrast to their findings in AC, their findings in VC are in agreement with the present observations, which seem to indicate a differential sensitivity of cortical regions towards different CMS paradigms, as also discussed previously [13, 77]. While so far not much data exists to support the crucial role of AC in stress exposure and response, the existing evidence does indicate that AC is sensitive to CMS exposure.

Findings indicating differential and contrasting microstructural alterations seem to fit well with the immense heterogeneity of depression symptoms. With this in mind, one might speculate to what extent the animals in our groups respond differently to the CMS paradigm. Certainly, the resilient animals respond differently than those that develop anhedonic behavior. It is therefore not unlikely that within-group CMS response might vary. Our data support this line of thought since broader CI is seen in both of the stress groups in both d-MRI and histological data potentially indicating heterogenous microstructural alterations in CMS model of depression [39].

A link between depression and motor symptoms is known from Parkinson’s disease which is often preceded by depression prior to the onset of the motor symptoms that allows clinical diagnosis [41]. Janakiraman et al. (2016) have shown significantly lower dopamine and serotonin as well as a marked motor impairment in a depression and preclinical Parkinson’s disease model [78]. In relation to these findings, another observation of the present study is higher *W*_T_ in MC of the anhedonic group in comparison to control. Elevated *W*_T_ may be caused by increased density of parallel axons in agreement with our histology where significantly higher axonal density was found in MC of the resilient group. Based on this observation, future studies might consider to include white matter markers such as kurtosis fractional anisotropy [79] or white matter tract integrity (WMTI) analysis which due to recent development can be performed based on fast kurtosis data sets [48, 80, 81]. A significantly lower cortical thickness and higher axonal density in MC in the resilient group also support the elevated *W*_T_ of the MC. Cortical thining is reported by a range of clinical and postmortem studies in case of depression and similar depressive disorders [22, 23, 82]. These findings are also in agreement with the significantly higher FA of MC in the resilient group and a modest increase in MR based neurite density. While significantly increased FA was found in the MC of the resilient group, however, is not specific for any tissue microstructure, whereas the neurite density model parameters are specific to the tissue microstructure and potentially reveals the biological underpinnings of changes in DTI and kurtosis parameters. Nevertheless, the histological data reveals thinner MC and higher axonal fraction in the MC which might explain the observed increase in FA in MC [67].

We found that diffusion tensor parameter (FA) and neurite density model parameter, *D*_eff_ showed significant microstructural alterations in cortical ROIs as was also supported by immunohistochemistry. However, validation of MR findings is still limited and so far, only FA was compared to fiber orientations using structure tensor analysis of histological data [83–86].

Although a limitation of the present study is to sample sub regions of the cortical ROIs from the d-MRI data to get matching ROIs in the immunohistological data, however there was no significant correlation between the d-MRI metrics and axonal and dendritic fraction of the immunohistological images. Nonetheless, the observed microstructural alterations in AC and MC suggest a neural basis for underlying behavioral changes related to MC and AC. Furthermore, our findings imply that cortical regions are also sensitive towards depression, perhaps due to allostatic regulation in cortical and sub-cortical regions of the brain. [12, 87, 88]. These cortical findings call for further investigations of behavioral changes associated with cortical areas, and may also provide potential new avenues for interpreting visual and auditory fear conditioning paradigms, as well as provide novel therapeutics targets for depression and similar mental disorders.

## Conclusion

The present study emphasized the role of AC and MC in the CMS induced depression model. The biophysical model parameter, *D*_eff_ and diffusion tensor parameter, FA demonstrated a potential to reveal microstructural alterations in this depression model.d-MRI. The present study indicates that the extended neural circuitry of depression may include AC and MC and also suggests performing behavioral studies focused on auditory and motor function in depressed rats. The clinically feasible d-MRI metrics employed in the present study may be useful in diagnosis and/or for monitoring treatment outcome in depression and similar disorders.

## Supporting information

S1 Fig (a) Mean kurtosis (MK), (b) Axial kurtosis (AK) and (c) Radial kurtosis (RK) data as mean ± confidence interval (CI) from MC, VC, AC and SC regions of the brain from control, anhedonic and resilient group. Linear mixed model regression analysis was performed in Matlab. No significant alteration was observed in any ROIs of the stress group with all the three kurtosis parameters in comparison to control.
(TIF)

S2 Fig Dendritic density % data as mean ± confidence interval from MC, VC, AC and SC regions of the brain from control, anhedonic and resilient group. No significant alteration was observed in any region of the stress groups in comparison to control.
(TIF)

S1 File Average effect size (ES) and confidence interval (CI) of axonal density (%), dendritic density (%), and cortical thickness form MC, SC, AC, and VC region of the brain from control, anhedonic, and resilient group. (XLSX)
