# Supplementary figures and images for: Differential microstructural alterations in rat cerebral cortex in a model of chronic mild stress depression
Source: PLoS One. 2018 Feb 12;13(2):e0192329. doi: 10.1371/journal.pone.0192329 (PMC5809082; doi:10.1371/journal.pone.0192329)

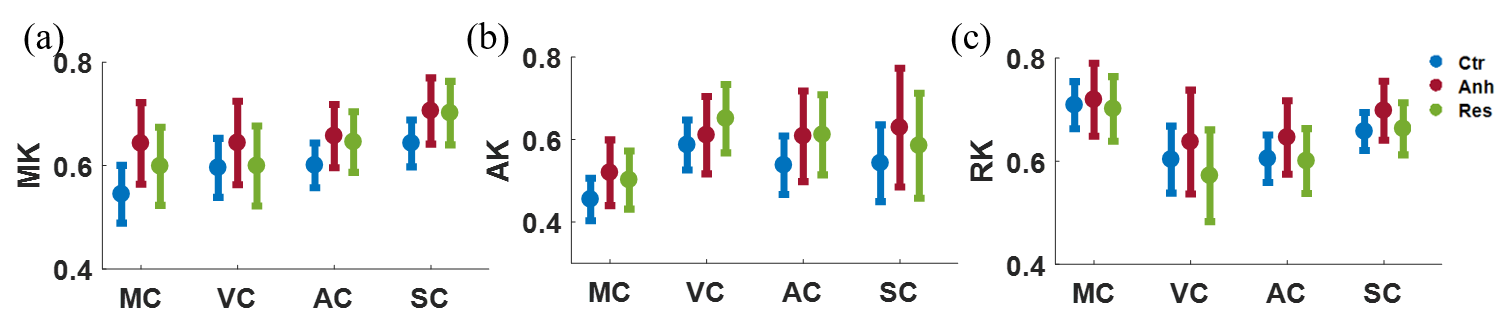

Supplement: S1 Fig — (a) Mean kurtosis (MK), (b) Axial kurtosis (AK) and (c) Radial kurtosis (RK) data as mean ± confidence interval (CI) from MC, VC, AC and SC regions of the brain from control, anhedonic and resilient group. Linear mixed model regression analysis was performed in Matlab. No significant alteration was observed in any ROIs of the stress group with all the three kurtosis parameters in comparison to control. (TIF) [file pone.0192329.s001.tif]

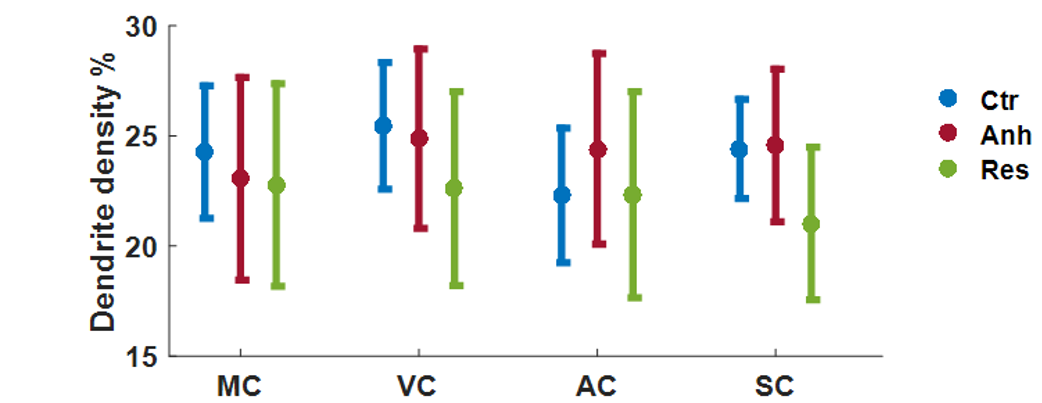

Supplement: S2 Fig — No significant alteration was observed in any region of the stress groups in comparison to control. (TIF) [file pone.0192329.s002.tif]
